# Single‐Cell Transcriptomic Analysis Suggests That JUN May Regulate BAMBI to Promote Osteosarcoma Cell Migration and Invasion

**DOI:** 10.1155/humu/9261651

**Published:** 2026-05-27

**Authors:** Ning Song, Qiang Zhang, Junwei Du, Renbing Jiang

**Affiliations:** ^1^ Department of Bone and Soft Tissue Tumor and Melanoma, Cancer Hospital of Xinjiang Medical University, Urumqi, Xinjiang, China

**Keywords:** biomarker, intratumor heterogeneity, migration and invasion, osteosarcoma, single-cell transcriptomics

## Abstract

**Background:**

Distant metastasis of osteosarcoma (OS) is one of the major factors contributing to poor patient prognosis, and its underlying mechanisms remain incompletely understood. In this study, we employed single‐cell RNA sequencing (scRNA‐seq) to assess intratumoral heterogeneity and identify immune diagnostic and therapeutic markers, providing novel insights to guide targeted therapy.

**Methods:**

Key cell clusters were identified using InferCNV analysis and combined with differential gene expression analysis, pseudotime trajectory analysis, transcription factor regulatory network construction, and functional enrichment analysis; signaling pathways closely associated with OS metastasis were screened and subsequently validated through the GSE152048 dataset and in vitro functional experiments.

**Results:**

In this study, a total of 10 cell clusters were identified. Immune cells exhibited higher infiltration levels in the metastatic group. Further dimensionality reduction and clustering of malignant osteoblastic cells revealed five subpopulations, among which the metastatic C1 and C3 subclusters exhibited more pronounced malignant features. The results showed that BAMBI was specifically downregulated during the transition from nonmetastatic to metastatic states. Further analysis indicated that JUN may maintain subcluster phenotypic stability by regulating BAMBI expression. Moreover, as a pseudoreceptor of the TGF‐*β* signaling pathway, the downregulation of BAMBI can promote pathway activation, thereby enhancing the migration and invasion potential of OS cells. Furthermore, the results were validated in the GSE152048 dataset and cell experiments.

**Conclusion:**

This study reveals that during the progression of OS from a nonmetastatic to a metastatic state, BAMBI expression is downregulated and may be regulated by JUN. The decreased expression of BAMBI leads to hyperactivation of the TGF‐*β* signaling pathway, thereby promoting the migration and invasion of OS cells. These findings provide potential immune‐related and molecular targets that could inform single cell–guided strategies for targeted therapy and overcoming resistance.

## 1. Introduction

Osteosarcoma is a highly malignant primary bone tumor characterized by aggressive invasiveness and a significant tendency for distant metastasis [1]. Currently, the standard treatment strategy for OS involves neoadjuvant chemotherapy, surgery and subsequent adjuvant chemotherapy [2]. Although the combination of surgery and chemotherapy has significantly improved the prognosis of patients with localized disease, with a 5‐year survival rate of 60%~70% [3, 4], the prognosis markedly worsens once metastasis occurs, with the 5‐year survival rate dropping to 20%~30% [5, 6]. Therefore, the high mortality associated with metastasis remains one of the most critical challenges in the treatment of OS.

A notable characteristic of OS is the high heterogeneity among different tumor cell populations within the tumor, as well as significant biological differences observed between patients [7]. Tumor heterogeneity is considered a critical factor influencing tumor progression, metastasis, and therapeutic response. However, studies investigating the role and underlying mechanisms of tumor heterogeneity in OS metastasis remain limited. Systematic analysis of the transcriptional landscape of OS holds promise for elucidating its tumor heterogeneity and progression mechanisms. This, in turn, could provide a theoretical basis for developing more targeted therapeutic strategies to effectively combat this highly aggressive tumor type.

Compared with conventional bulk sequencing technologies, scRNA‐seq enables the characterization of genetic and functional heterogeneity within tumors at single‐cell resolution, facilitating the identification of rare cell subpopulations and the reconstruction of intercellular interaction networks [8]. Recent studies comparing the cellular composition of metastatic and nonmetastatic OS microenvironments have revealed that metastatic OS exhibit pronounced immune evasion features compared with their nonmetastatic counterparts [9]. Existing studies have predominantly employed scRNA‐seq to characterize the cellular composition of OS; however, most of these investigations remain limited to static descriptions of the tumor microenvironment (TME) components, lacking systematic exploration of the dynamic evolutionary mechanisms of tumor cells during metastasis. In this study, we utilized the public dataset GSE250015 and applied scRNA‐seq coupled with bioinformatics analyses to identify rare cell subpopulations significantly associated with OS metastasis. We systematically characterized dynamic regulatory networks in metastatic and nonmetastatic OS and validated their impact on tumor migration and invasion potential at the cellular level, elucidating key mechanisms driving tumor migration and invasion. These findings not only provide a single‐cell level theoretical basis for understanding OS migration and invasion but also lay the foundation for developing novel therapeutic strategies targeting the TME.

## 2. Materials and Methods

### 2.1. Data Acquisition

The scRNA‐seq data used in this study were obtained from the Gene Expression Omnibus (GEO) public database (https://www.ncbi.nlm.nih.gov/), under the Accession Number GSE250015. This dataset contains single‐cell transcriptomic data derived from tumor tissues of two patients with primary OS, including one case with a metastatic lesion exhibiting distant metastasis and one case with a nonmetastatic lesion without evidence of metastasis. To ensure the reliability of the analysis results, the dataset GSE152048 was selected as the validation set, which includes primary samples (BC2 and BC22) and metastatic samples (BC10 and BC17).

### 2.2. Clustering and Dimensionality Reduction of scRNA‐seq Data

scRNA‐seq data analysis was performed in the R software environment (Version 4.4.2), and data preprocessing was conducted using the Seurat package (Version 5.2.1). To ensure data quality, low‐quality cells were filtered out. Genes expressed in fewer than three cells were excluded. Cells with fewer than 400 or more than 4000 detected genes, or with mitochondrial gene content exceeding 15%, were removed. The NormalizeData function was used to perform log‐normalization (LogNormalize, scale.factor = 10,000) on the raw count matrix. Highly variable genes (Top 2000) were identified using the FindVariableFeatures function. Subsequently, linear dimensionality reduction was performed using the ScaleData and RunPCA functions. To integrate multiple samples and eliminate batch effects, data integration was performed using the FindIntegrationAnchors and IntegrateData functions based on the CCA method. Subsequently, a nearest neighbor graph was constructed based on the PCA results. Unsupervised clustering of major cell populations was performed using the FindNeighbors and FindClusters functions in the Seurat package. t‐SNE was applied for visualization. Finally, cluster‐specific marker genes were identified using the FindAllMarkers function. Cell type annotation was performed by integrating canonical marker genes from the literature with SingleR‐based annotation [9, 10].

### 2.3. Copy Number Variation (CNV) Analysis

To assess genomic instability across different OS cell subtypes, we performed chromosomal CNV inference on single‐cell RNA sequencing data using the R package inferCNV (Version 1.22.0). We extracted the osteoblastic cell subtypes annotated within the tumor samples as the target population for analysis, whereas endothelial cells derived from normal tissues were selected as the reference population. Subsequently, an integrated expression matrix was constructed alongside a corresponding cell annotation table. Tumor cells were annotated as Clusters C1–C5 based on Seurat clustering results, whereas the reference group was labeled as “normal.” Gene chromosomal location information was retrieved from the Ensembl BioMart database. Only genes uniquely annotated and located on Chromosomes 1 through 22 were retained to construct the gene ordering file required for CNV analysis. The CreateInfercnvObject function was used with the cutoff parameter set to 0.1. Subsequently, the analysis pipeline was executed by calling the infercnv::run function, employing default parameters for denoising and HMM inference. This process computed the CNV status of each cell across different chromosomal regions. A CNV heatmap was generated for visualization, illustrating the differences in chromosomal CNV patterns among various cell subpopulations. To quantify CNV levels, the CNV score for each cell was calculated as the mean of the absolute values across columns in the CNV expression matrix. Differences in CNV scores between metastatic and nonmetastatic groups were compared within each cluster.

### 2.4. Pseudotime Analysis

Pseudotime analysis was performed using the Monocle R package (Version 2.34.0). The raw UMI count matrix was first extracted from the Seurat object and converted into a CellDataSet object. Genes expressed in at least 10 cells were retained, and highly variable genes were selected based on empirical dispersion estimates. Dimensionality reduction was then performed using the DDRTree method, and cells were ordered along the pseudotime trajectory. Differentially expressed genes (DEGs) along the pseudotime trajectory were identified by fitting smooth spline curves and applying multiple testing correction with a FDR threshold of < 0.01. Top‐ranked genes were subjected to hierarchical clustering combined with pseudotime ordering to visualize their dynamic expression patterns.

### 2.5. Transcription Factor Regulatory Network Analysis

In this study, transcription factor regulatory network analysis of osteoblastic cell subpopulations in OS was performed using the R package SCENIC (Version 1.3.1). First, target cell populations were selected based on Seurat clustering, and the corresponding expression matrix and cell annotation information were extracted. Subsequently, the SCENIC analysis pipeline was applied to construct gene coexpression networks. The RcisTarget step was performed using the hg19 motif database (cisTarget databases: hg19‐500 bp‐upstream‐7species and hg19‐tss‐centered‐10 kb‐7species) to identify regulons with enriched transcription factor binding motifs. The gene coexpression networks were computed using the runSCENIC function. Subsequently, the GENIE3 algorithm was employed to construct coexpression networks between transcription factors and their potential target genes, generating the initial regulatory network. Regulons were retained based on motif enrichment criteria (normalized enrichment score, NES > 3.0). The AUCell algorithm was used to calculate the activity scores of each regulon at the single‐cell level. Further analyses were performed to assess the regulatory specificity of these regulons across different cell types (metastatic vs. nonmetastatic cells). Finally, heatmaps and RSS plots were generated to identify key transcriptional regulators associated with tumor metastasis.

### 2.6. Cell Culture and Transfection

The human OS cell lines 143B, HOS, and SaOS2 used in this study were all purchased from ServiceBio (Wuhan, China). Among them, 143B and HOS cells were cultured in DMEM supplemented with 10% FBS and 1% P/S, whereas SaOS2 cells were maintained in McCoy′s 5A medium containing 10% FBS and 1% P/S. All cells were maintained in a humidified incubator at 37°C with 5% CO_2_. When the cells reached 70%–80% confluence, they were digested with 0.25% trypsin (Biosharp, China) and reseeded into 6‐well plates for subsequent transfection experiments. After 12 h of cell adherence, the cell density was adjusted according to the type of transfection: approximately 50% confluence for siRNA transfection and 70% confluence for plasmid transfection. Transfections were performed using Lipo6000 transfection reagent (Beyotime, China), with Opti‐MEM reduced‐serum medium (Beyotime, China) used to prepare the transfection complexes. After 6 h of transfection, the medium was replaced with fresh complete culture medium. Cells in the siRNA group were cultured for an additional 72 h posttransfection, whereas cells in the plasmid group were cultured for 48 h before collection for subsequent experiments. The siRNA sequences used in the functional experiments are provided in Table S1.

### 2.7. Transwell Migration and Invasion Assay

Cell migration and invasion abilities were assessed using Transwell chambers (Corning, United States). Log‐phase human OS cells (143B and SaOS2) were subjected to intervention treatment for 24 h. Subsequently, 500 *μ*L of serum‐free RPMI‐1640 medium (Gibco, United States) was added to both the upper and lower chambers of the Transwell apparatus, which was then incubated at 37°C for 2 h to hydrate and equilibrate the coated basement membrane. For the migration assay, the upper chamber membrane was not coated with Matrigel. In contrast, for the invasion assay, the membrane surface of the upper chamber was uniformly coated with Matrigel (BD Biosciences, United States) 2 h prior to the experiment. Cells were then collected and washed once with PBS, followed by centrifugation at 1500 rpm for 5 min. The cell pellet was resuspended in serum‐free medium and counted. The cell suspension was diluted to 3 × 10^5^ cells/mL, and 500 *μ*L was added to the upper chamber. The lower chamber was filled with 750 *μ*L of complete medium containing 10% FBS. The assembly was incubated at 37°C for 24 h to establish a chemotactic gradient, inducing cell migration or invasion. After incubation, the upper chamber was gently washed twice with prechilled PBS, and nonmigrated cells remaining on the upper surface of the membrane were carefully removed using a sterile cotton swab. Two hundred microliter of methanol was added to both the upper and lower chambers and fixed at room temperature for 10 min. After discarding the methanol, 200 *μ*L of 0.1% crystal violet staining solution was added to the upper chamber and stained at room temperature for 10 min. Following staining, the chambers were washed twice with PBS to remove excess dye. Five random fields of view were selected per Transwell chamber for microscopic imaging, and the number of cells that had migrated or invaded through the membrane was counted to assess the cells′ migratory or invasive capabilities.

### 2.8. Quantitative Real‐Time PCR (qRT‐PCR) Analysis

Total RNA was extracted from transfected cells using TriQuick Reagent (Solarbio, China) according to the manufacturer′s instructions. The extracted RNA was then reverse transcribed into cDNA using Evo M‐MLV Reverse Transcriptase (Accurate Biology, China) according to the manufacturer′s instructions. Subsequently, qRT‐PCR amplification was performed using PerfectStart Green qPCR SuperMix (+Dye II) (TransGen Biotech, China). *β*‐actin was used as the internal control gene to normalize the expression levels of target genes. The relative expression of target genes was calculated using the 2^−*ΔΔ*Ct^ method. The sequences of all primers are provided in Table S2.

### 2.9. Western Blotting (WB)

Whole‐cell lysates were extracted using RIPA lysis buffer (Powerful Biology, China). Proteins were separated by SDS‐PAGE (Biosharp, China) and subsequently transferred onto methanol‐activated PVDF membranes (BaiDai, United States). The membranes were blocked with 5% nonfat milk (Biosharp, China) at room temperature for 30 min, followed by incubation with primary antibodies overnight at 4°C. After washing three times with TBST buffer (Powerful Biology, China), the membranes were incubated with HRP‐conjugated secondary antibodies at room temperature for 30 min, followed by three additional washes with TBST. Protein bands were visualized using ECL chemiluminescent reagents (Powerful Biology, China). Images were captured, and the band intensities were quantified using AlphaView software. *β*‐actin was used as the internal loading control.

### 2.10. Transwell Migration and Invasion Assays

Cell migration and invasion abilities were assessed using Transwell chambers (Corning, United States). Human OS cells (143B and SaOS2) in the logarithmic growth phase were subjected to the indicated treatments for 24 h. Subsequently, 500 *μ*L of serum‐free RPMI‐1640 medium (Gibco, United States) was added to both the upper and lower chambers of the Transwell inserts and incubated at 37°C for 2 h to hydrate and equilibrate the precoated basement membrane.

For the migration assay, the upper chamber membranes were left uncoated with Matrigel, whereas for the invasion assay, the upper membranes were evenly coated with Matrigel (BD Biosciences, United States) 2 h prior to the experiment. Subsequently, the cells were harvested, washed once with PBS, centrifuged at 1500 rpm for 5 min, resuspended in serum‐free medium, and counted. The cell suspension was then adjusted to a concentration of 3 × 10^5^ cells/mL, with 500 *μ*L of the suspension added to the upper chamber and 750 *μ*L of complete medium containing 10% FBS added to the lower chamber. The chambers were incubated for 24 h to establish a chemotactic gradient that induced cell migration or invasion. At the end of incubation, the upper chambers were rinsed twice with precooled PBS, and nonmigrated cells on the upper surface of the membranes were gently removed using sterile cotton swabs. Both the upper and lower chambers were then treated with 200‐*μ*L methanol at room temperature for 10 min to fix the cells. After discarding the methanol, 200 *μ*L of 0.1% crystal violet solution was added to the upper chambers for staining at room temperature for 10 min. Excess dye was removed by washing twice with PBS. For quantification, five random microscopic fields were selected from each Transwell insert, imaged, and the number of migrated or invaded cells was counted to evaluate the migration and invasion capacities.

### 2.11. Statistical Analysis

Statistical analyses were performed using R software (Version 4.3.1) and GraphPad Prism (Version 9.0). For comparisons of continuous variables such as gene expression, an unpaired Student′s *t*‐test was used. Pearson′s correlation coefficient was applied to evaluate the relationship between two variables. All data are presented as mean ± SD. All statistical tests were two‐sided, and *p* values less than 0.05 were considered statistically significant.

## 3. Results

### 3.1. Establishment of a Single‐Cell Atlas of OS

**Figure 1:**
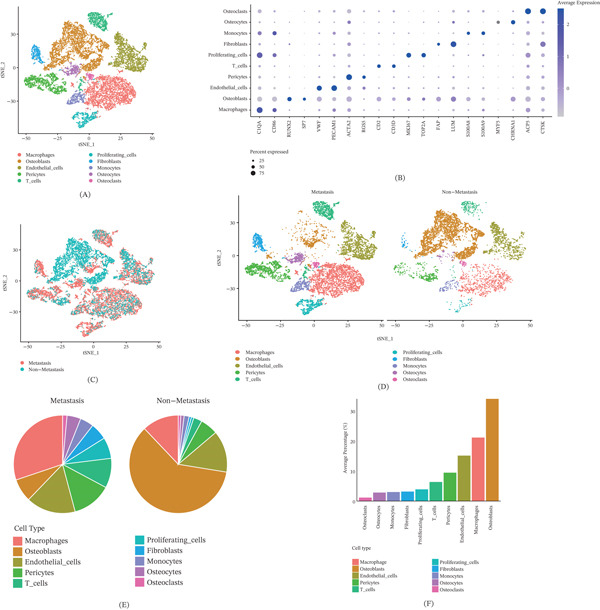
Comparative single‐cell atlas analysis of metastatic and nonmetastatic OS lesions. (A) t‐SNE plot showing 10 distinct cell clusters identified in OS lesions, (B) expression of 20 signature genes across the 10 cell clusters, (C) distribution of the 10 cell clusters in metastatic versus nonmetastatic OS lesions displayed by t‐SNE, (D) t‐SNE visualization of metastatic and nonmetastatic OS lesions, (E) pie charts depicting the proportional distribution of cell types in metastatic and nonmetastatic OS lesions, and (F) average proportions of different cell types in metastatic and nonmetastatic OS lesions.

We performed scRNA‐seq analysis on dataset GSE250015 retrieved from the GEO database to investigate the single‐cell atlas of metastatic and nonmetastatic OS lesions. After rigorous quality control and filtering, a total of 15,551 cells and 41,992 genes were identified. Among them, metastatic OS lesions comprised 9126 cells expressing 20,745 genes, whereas nonmetastatic OS lesions included 6425 cells with 21,247 genes detected. Dimensionality reduction followed by clustering analysis was conducted for data visualization, through which 10 major cell clusters were identified. Cell type annotation of each cluster was performed based on their gene expression profiles and known canonical marker genes (Figure 1A–D), as follows: macrophages (C1QA, CD86), osteoblasts (RUNX2, SP7), endothelial cells (VWF, PECAM1), pericytes (ACTA2, RGS5), T cells (CD2, CD3D), proliferating cells (MKI67, TOP2A), fibroblasts (FAP, LUM), monocytes (S100A8, S100A9), osteocytes (MYF5, CHRNA1), and osteoclasts (ACP5, CTSK). Figure 1E visually depicts the relative proportions of cell types across different samples. Notably, macrophages constituted the largest fraction in the metastatic group, followed by endothelial cells and T cells. In the nonmetastatic group, osteoblasts accounted for a significantly higher proportion compared with other cell types, suggesting a more prominent role in tissue composition under nonmetastatic conditions. Figure 1F presents the average proportions of different cell types, with osteoblasts showing the most significant difference between groups.

### 3.2. Heterogeneity of Osteoblast Subpopulations and Their Distribution Characteristics

**Figure 2:**
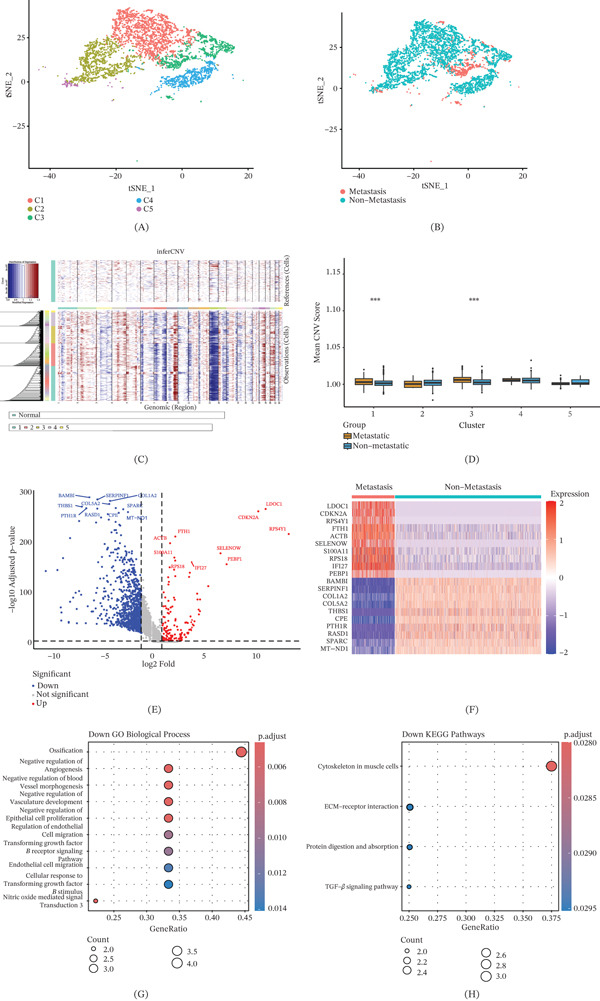
Integrative analysis of CNV inference and differential expression reveals metastatic potential of osteoblast subpopulations in OS. (A) t‐SNE analysis identifying five major osteoblast subclusters, (B) t‐SNE plot showing osteoblasts from metastatic and nonmetastatic OS lesions, (C) heatmap illustrating CNV profiles of osteoblast subclusters, (D) boxplot comparing CNV scores between osteoblast subclusters from metastatic and nonmetastatic OS lesions, (E) volcano plot of DEGs between Clusters C1 and C3 in metastatic versus nonmetastatic osteoblasts, (F) heatmap of the Top 10 DEGs between Clusters C1 and C3 in metastatic versus nonmetastatic osteoblasts, (G) GO enrichment analysis of the Top 10 downregulated DEGs, and (H) KEGG pathway enrichment analysis of the Top 10 downregulated DEGs ( ^∗^ *p* < 0.05,  ^∗∗^ *p* < 0.01,  ^∗∗∗^ *p* < 0.001).

Osteoblasts represent the predominant cell type in OS. To further explore the heterogeneity of osteoblast subpopulations, we performed a second round of dimensionality reduction analysis, which identified five distinct subclusters (Figure 2A). Figure 2B shows the distribution of osteoblasts derived from metastatic and nonmetastatic samples. OS exhibits highly complex genomic instability, characterized by widespread and recurrent somatic copy number alterations and structural rearrangements, whereas the frequency of recurrent point mutations in protein‐coding genes remains relatively low. These findings suggest that key oncogenic events driving the initiation and progression of this malignancy may predominantly arise from gene abnormalities associated with somatic copy number alterations [11]. Based on this, we performed CNV analysis on the five osteoblast subclusters using the inferCNV algorithm to further evaluate their potential malignant characteristics (Figures 2C and S1). Meanwhile, CNV inference was separately performed on single‐cell samples derived from metastatic and nonmetastatic OS lesions to compare the genomic instability of osteoblast subclusters between different lesions, thereby uncovering their potential contributions to tumor heterogeneity. The results showed that clusters C1 and C3 exhibited pronounced CNV abnormalities, suggesting their potential involvement in tumor invasion and metastasis (Figure 2D). We further analyzed the DEGs between Clusters C1 and C3 in metastatic and nonmetastatic OS samples. Significantly DEGs were identified through differential expression analysis and visualized using volcano and heatmaps (Figure 2E,F), highlighting the Top 10 representative DEGs. Subsequently, functional enrichment analyses—including Gene Ontology (GO) and Kyoto Encyclopedia of Genes and Genomes (KEGG) pathway analyses—were performed on the Top 10 downregulated DEGs in metastatic samples (Figure 2G,H). These genes were primarily enriched in processes such as ossification, negative regulation of angiogenesis, and the TGF‐*β* signaling pathway.

### 3.3. Potential Role of BAMBI as a Key Gene in OS Metastasis

**Figure 3:**
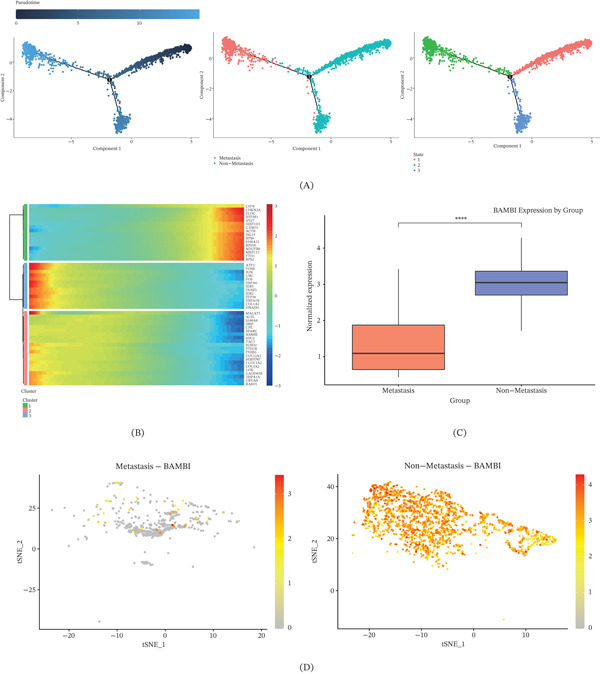
Pseudotime analysis reveals the potential role of BAMBI as a key gene in OS metastasis. (A) Distribution of metastatic and nonmetastatic cells along the trajectory and their pseudotime progression, with colors from dark to light indicating cells progressing toward the terminal state, (B) heatmap of differentially expressed genes identified by pseudotime trajectory analysis, (C) boxplot comparing BAMBI expression between metastatic and nonmetastatic cell clusters, and (D) t‐SNE plots illustrating the spatial distribution and differential expression of BAMBI in metastatic and nonmetastatic cell clusters ( ^∗^ *p* < 0.05,  ^∗∗^ *p* < 0.01,  ^∗∗∗^ *p* < 0.001).

To further investigate the dynamic changes of Clusters C1 and C3 during OS metastasis, we performed pseudotime analysis using Monocle 2 and constructed cellular differentiation trajectories to elucidate the potential developmental pathways involved in OS metastasis (Figure 3A). The results demonstrated that cells gradually progressed along the pseudotime trajectory, with nonmetastatic cells predominantly localized at the early differentiation stages, whereas metastatic cells were mainly distributed at the terminal end of the trajectory, suggesting the presence of distinct differentiation endpoints or cellular states associated with metastasis. Further, clustering analysis of DEGs during the transition from State 1 to State 2 along the pseudotime trajectory was performed using heatmaps, revealing multiple dynamic gene expression modules. These modules suggest that the identified genes may play crucial roles in regulating late‐stage cell fate decisions (Figure 3B). Further comparison of BAMBI expression between metastatic and nonmetastatic cells revealed that BAMBI was significantly upregulated in nonmetastatic cells, suggesting a potential inhibitory role of BAMBI in tumor metastasis (Figure 3C). In the t‐SNE visualization, BAMBI exhibited sparse expression in metastatic cells, whereas it was broadly and highly expressed in nonmetastatic cells, consistent with the differential expression results and spatial distribution patterns (Figure 3D).

### 3.4. JUN May Regulate OS Cell Metastatic Potential via Modulation of BAMBI Expression

**Figure 4:**
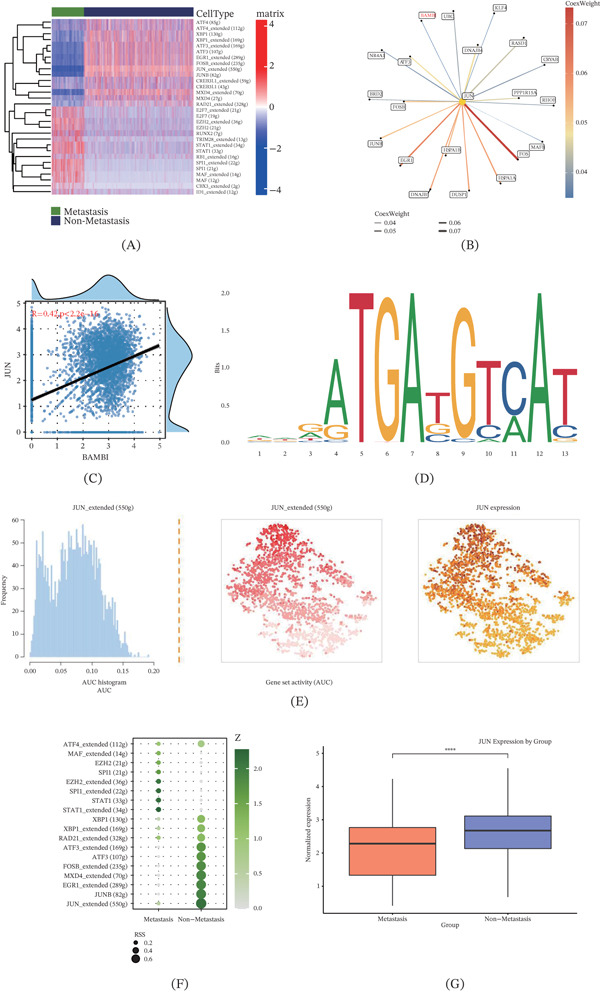
JUN may regulate OS osteoblast metastasis through modulation of BAMBI expression. (A) Heatmap showing transcription factor activity enrichment between metastatic and nonmetastatic OS lesions, (B) transcriptional regulatory network highlighting JUN as a key transcription factor, (C) correlation analysis between JUN and BAMBI expression, (D) predicted JUN binding sequences in the BAMBI promoter region based on the JASPAR database, (E) distribution of JUN activity at the single‐cell level assessed using the AUCell algorithm, (F) bubble plot illustrating differential transcription factor activity between metastatic and nonmetastatic cell clusters, and (G) box plot showing JUN expression differences between metastatic and nonmetastatic clusters ( ^∗^ *p* < 0.05,  ^∗∗^ *p* < 0.01,  ^∗∗∗^ *p* < 0.001).

To further investigate the upstream regulatory mechanisms of BAMBI, we applied the SCENIC analysis pipeline to systematically identify transcription factors and their regulatory networks in osteoblasts. Several transcription factors with regulatory activity were identified, among which 20 were significantly enriched in osteoblast subpopulations. Analysis revealed that JUN, a principal member of the activator protein‐1 (AP‐1) transcription factor family, serves as a central regulator within the network and may have a pivotal regulatory relationship with BAMBI (Figure 4A,B), suggesting its critical role in the pathogenesis of OS. Moreover, Pearson correlation analysis demonstrated a moderate positive correlation between JUN and BAMBI expression (*R* = 0.42, *p* < 0.001), supporting their potential regulatory linkage within this axis (Figure 4C). To further explore whether JUN may directly regulate BAMBI, cis‐element analysis was performed using the JASPAR database to predict potential JUN binding sites within the BAMBI promoter region, with a relative profile score threshold of 85%. The results identified multiple potential JUN‐binding motifs, among which the most significant motif, TGCCTCA, was located at the relative position of 830–836 bp. These findings support JUN as a putative upstream regulator of BAMBI (Figure 4D). Subsequently, we quantified the activity of transcription factors using the AUCell method. The results revealed that JUN exhibited significantly higher activity (AUC score) in nonmetastatic cells compared with metastatic cells, indicating its robust transcriptional regulatory potential (Figure 4E,F). Further comparison of JUN expression levels between metastatic and nonmetastatic samples revealed a significant downregulation of JUN in metastatic samples (Figure 4G).

Single‐cell transcriptome analysis was performed on four samples from the validation dataset GSE152048, identifying 10 major cell clusters. Cell types were annotated based on canonical marker genes as follows: osteoblasts (RUNX2, COL1A1), T cells (CD2, CD3D), pericytes (ACTA2, RGS5), proliferating cells (MKI67, TOP2A), osteoclasts (ACP5, MMP9), endothelial cells (VWF, PECAM1), monocytes (CD14, FCGR3A), fibroblasts (DCN, COL10A1), macrophages (C1QA, C1QB), and B cells (CD79A, JCHAIN) (Figure S2A–D). Osteoblasts were further subjected to secondary dimensionality reduction and clustering (Figure S3A,B), and inferCNV analysis was applied to assess their genomic CNVs, confirming their potential malignant phenotype (Figure S3C,D). The analysis showed that BAMBI and JUN expression was lower in the metastatic osteoblast subcluster (Figures S3E and S4). Correlation analysis between JUN and BAMBI expression indicated a weak positive relationship (Figure S3F).

### 3.5. The Effects of BAMBI and Its Upstream Regulator JUN on the Invasion and Metastasis of OS Cells

**Figure 5:**
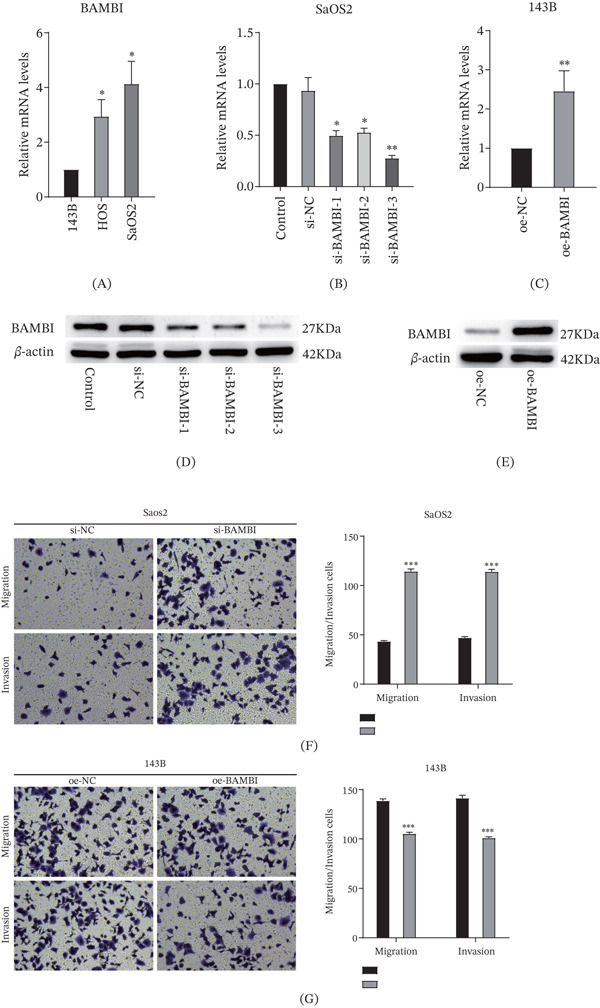
In vitro validation of BAMBI expression in OS cell lines. (A) Comparison of BAMBI expression levels among three cell lines, showing the highest expression in SaOS2 cells and the lowest in 143B cells; (B–E) qRT‐PCR and Western blot analyses demonstrating successful silencing of BAMBI in SaOS2 cells and overexpression in 143B cells; (F) Transwell migration and invasion assays following BAMBI knockdown in SaOS2 cells. (G) Transwell migration and invasion assays following BAMBI overexpression in 143B cells ( ^∗^ *p* < 0.05,  ^∗∗^ *p* < 0.01,  ^∗∗∗^ *p* < 0.001).

To investigate the potential role of BAMBI in OS metastasis, we first compared its mRNA expression levels in three OS cell lines—143B, HOS, and SaOS2—using qRT‐PCR analysis. The results showed that BAMBI expression was relatively high in SaOS2 cells, whereas it was lower in 143B cells (Figure 5A). Based on the above results, SaOS2 and 143B cell lines were selected as representative models. At the early stage of the experiments, BAMBI‐targeting siRNAs and overexpression plasmids were constructed and transfected into cells. Preliminary validation demonstrated that si‐BAMBI effectively downregulated BAMBI expression, whereas the BAMBI overexpression vector achieved stable upregulation. Therefore, both were employed for subsequent functional assays (Figure 5B–E). Transwell migration and invasion assays demonstrated that BAMBI knockdown significantly enhanced the migratory and invasive capacities of both SaOS2 and 143B cells, whereas BAMBI overexpression markedly suppressed these abilities (Figure 5F,G).

**Figure 6:**
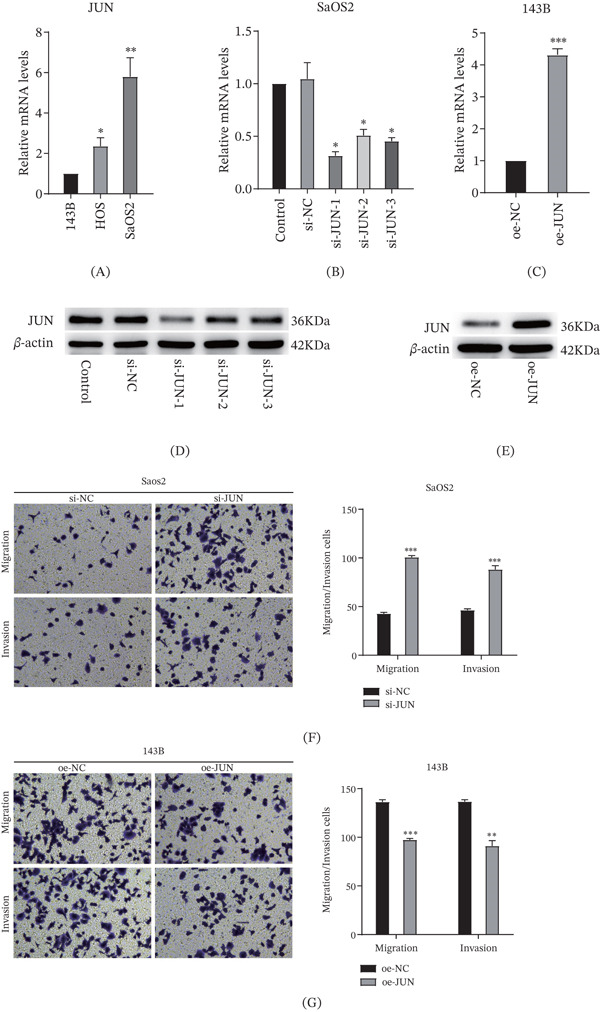
In vitro validation of JUN expression in OS cell lines. (A) Comparison of JUN expression levels among the three cell lines, showing the highest expression in SaOS2 cells and the lowest in 143B cells; (B–E) qRT‐PCR and Western blot analyses demonstrating successful silencing of JUN in SaOS2 cells and overexpression in 143B cells; (F) Transwell migration and invasion assays following JUN knockdown in SaOS2 cells. (G) Transwell migration and invasion assays following JUN overexpression in 143B cells ( ^∗^ *p* < 0.05,  ^∗∗^ *p* < 0.01,  ^∗∗∗^ *p* < 0.001).

Additionally, we assessed the expression levels of JUN, a transcription factor coexpressed with BAMBI. qRT‐PCR results showed that JUN expression was higher in SaOS2 cells with elevated BAMBI levels, whereas it was relatively lower in 143B cells with reduced BAMBI expression (Figure 6A). We further constructed and transfected JUN‐targeting siRNAs and overexpression plasmids, and validated their efficacy by assessing transfection efficiency. The results demonstrated that si‐JUN and the overexpression vector effectively knocked down and upregulated JUN expression, respectively (Figure 6B–E). Subsequent Transwell assays demonstrated that JUN knockdown significantly promoted cell migration and invasion, whereas JUN overexpression inhibited these abilities (Figure 6F,G).

### 3.6. Functional Correlation Analysis of JUN and BAMBI in OS Cell Migration and Invasion

**Figure 7:**
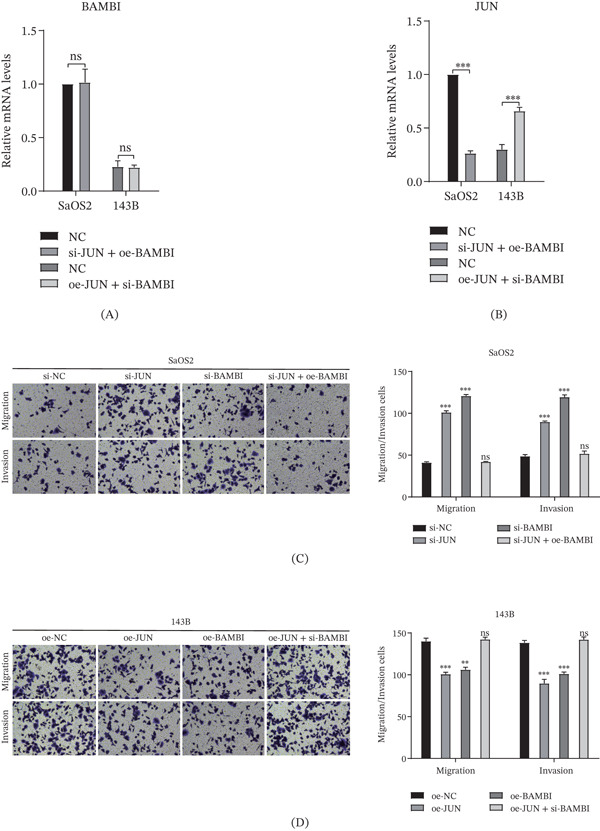
In vitro functional validation of JUN and BAMBI in OS cells. (A) qRT‐PCR analysis of BAMBI mRNA levels in SaOS2 and 143B cells; (B) qRT‐PCR analysis of JUN mRNA levels in SaOS2 and 143B cells; (C) Transwell migration and invasion assays of SaOS2 cells treated with si‐NC, si‐JUN, si‐BAMBI, or si − JUN + oe − BAMBI; (D) Transwell migration and invasion assays of 143B cells treated with oe‐NC, oe‐JUN, oe‐BAMBI, or oe − JUN + si − BAMBI ( ^∗^ *p* < 0.05,  ^∗∗^ *p* < 0.01,  ^∗∗∗^ *p* < 0.001, ns, not significant).

To elucidate the regulatory effect of the transcription factor JUN on BAMBI expression, we performed JUN overexpression and knockdown in SaOS2 and 143B cells, followed by assessment of BAMBI mRNA and protein levels. qRT‐PCR and WB analyses revealed that JUN overexpression significantly upregulated BAMBI expression, whereas JUN knockdown markedly downregulated BAMBI levels, suggesting that JUN may positively regulate BAMBI transcription and translation (Figure 7A,B). Further evaluation of cellular behavior using Transwell migration and invasion assays demonstrated that overexpression of either JUN or BAMBI significantly inhibited OS cell migration and invasion, whereas knockdown of either gene markedly enhanced these capabilities. Notably, under the conditions of JUN overexpression or knockdown, concomitant modulation of BAMBI expression significantly altered the migration and invasion phenotypes: BAMBI knockdown partially rescued migration and invasion abilities in JUN‐overexpressing cells, whereas BAMBI overexpression markedly suppressed migration and invasion in JUN‐knockdown cells (Figure 7C,D). These findings suggest that the effects of JUN on OS cell migration and invasion may be dependent on BAMBI expression.

## 4. Discussion

OS is the most common primary malignant bone tumor, characterized by high local invasiveness and a strong propensity for distant metastasis. Studies have shown that approximately 15%–20% of OS patients present with distant metastasis at the time of diagnosis, with the lungs being the most common metastatic site [12, 13]. Despite recent advances in the comprehensive treatment of OS, the overall prognosis for patients with metastatic or recurrent disease remains poor [14].

**Figure 8:**
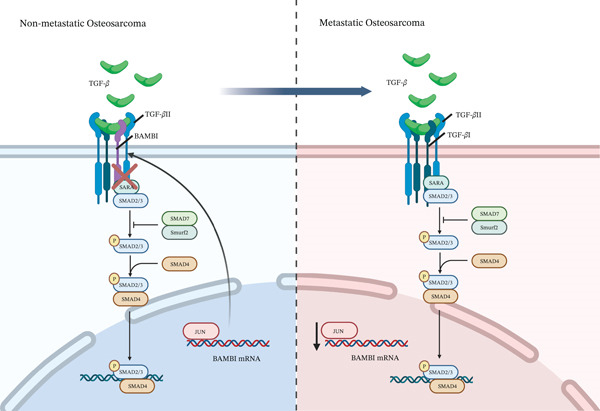
Proposed mechanistic model of TGF‐*β* signaling alterations in nonmetastatic and metastatic OS. In nonmetastatic OS (left), TGF‐*β* binds to its receptor complex, activating downstream signaling programs. In metastatic OS (right), the transcription factor JUN promotes the expression of the decoy receptor BAMBI, which competitively binds to the TGF‐*β* receptor complex, blocking the canonical TGF‐*β* signaling pathway and thereby promoting tumor metastasis.

Therefore, an in‐depth investigation into the molecular mechanisms underlying OS metastasis may provide a theoretical basis for developing more effective therapeutic strategies and holds significant clinical value in improving patient survival and quality of life. In this study, 10 distinct cellular clusters were identified based on single‐cell transcriptomic data, and key regulatory factors closely associated with OS metastasis were further screened and characterized. Among them, BAMBI showed specifically low expression in osteoblastic cells of Clusters 1 and 3, which possess strong metastatic potential, suggesting that BAMBI may play a critical role in promoting OS progression and migration and invasion. Further pathway enrichment and mechanistic studies indicate that BAMBI may promote OS migration and invasion by modulating the activation of the TGF‐*β* signaling pathway and facilitating EMT in tumor cells. Functional experiments demonstrated that inhibition of BAMBI expression significantly enhanced the migration and invasion capabilities of OS cells, suggesting that BAMBI may play a suppressive role in the migration and invasion process. Notably, this study also identified that BAMBI expression may be regulated by the upstream transcription factor JUN, suggesting the existence of a complex transcriptional regulatory network involved in OS metastasis and providing a theoretical basis for subsequent targeted interventions. A schematic model of the transition from nonmetastatic to metastatic OS is proposed in Figure 8.

The sustained activation of the AP‐1 complex plays a critical role in cellular transformation and tumorigenesis, and is commonly expressed across various types of human cancers [15–17]. Among them, JUN is one of the core components of the AP‐1 complex and widely participates in regulating gene expression involved in cell proliferation, differentiation, migration, apoptosis, inflammation, and tumorigenesis [18–21]. Animal model studies have shown that knockdown of c‐Jun significantly increases mortality risk and accelerates tumor initiation and progression [22]. Previous studies have demonstrated that the c‐Jun/CAP2 axis promotes gastric cancer metastasis by regulating M2 macrophage polarization and the TGF‐*β*1 feedback loop; meanwhile, rosmarinic acid B can target CAP2 to inhibit this process, suggesting that CAP2 may serve as a potential therapeutic target [23]. Additionally, SMAD3 and SMAD4 can synergistically regulate gene expression together with c‐Jun/c‐Fos, with crosstalk occurring between SMAD and MAPK/JNK signaling pathways at AP‐1 binding promoter regions. The RAS‐ERK/MAPK kinase pathway may cooperate with TGF‐*β* signaling to jointly promote EMT as well as the invasive and metastatic capabilities of tumor cells from various tissue origins [24, 25]. Previous studies have found that c‐Jun plays a key regulatory role during the differentiation of MSCs into the osteogenic lineage. Its expression is significantly upregulated throughout osteogenic differentiation, whereas knockdown of c‐Jun markedly reduces ALP activity and the expression of osteogenic marker genes, indicating that c‐Jun is an important transcription factor for osteogenic differentiation [26, 27]. These findings further support the hypothesis proposed in this study that JUN may play a crucial role in regulating OS invasion and metastasis.

BAMBI is a transmembrane glycoprotein characterized by an extracellular ligand‐binding domain structurally similar to that of TGF‐*β*RI, but lacking an intracellular kinase domain. Therefore, BAMBI is considered a negative regulatory pseudoreceptor of the TGF‐*β* signaling pathway. In hepatocellular carcinoma studies, PPARGC1A has been shown to regulate BAMBI expression by inhibiting the WNT/*β*‐catenin signaling pathway [28–30]. Subsequently, through the BAMBI/TGF‐*β*/SMAD axis, it modulates ACSL5 expression, ultimately affecting ROS levels and ferroptosis processes [31]. Studies have shown that BAMBI can inhibit TGF‐*β* signaling, thereby reducing the tumor infiltration capacity of MDSCs and attenuating their immunosuppressive effects. Delivery of BAMBI to the TME via adeno‐associated virus (AAV‐Bambi) has been shown to enhance the antitumor effects of radiotherapy alone as well as its synergistic effects with immunotherapy. Notably, the combination of AAV‐Bambi and radiotherapy not only improved local tumor control but also effectively suppressed distant metastasis, demonstrating promising potential for clinical translation [32]. In OS research, exogenous overexpression of BAMBI has been found to inhibit the activation of the TGF‐*β* signaling pathway, thereby suppressing TGF‐*β*–induced EMT, as well as cell proliferation, migration, and invasion. Conversely, knockdown of BAMBI enhances the pathway′s activity, suggesting that TGF‐*β*–mediated EMT may serve as a potential therapeutic target for OS [33]. Previous studies have shown that VAV1 regulates the growth of cutaneous T‐cell lymphoma through the BAMBI/BMF signaling pathway. Silencing of VAV1 induces apoptosis and suppresses proliferation, accompanied by the upregulation of the Bcl‐2 modifying factor and the downregulation of BAMBI [34]. Additionally, some studies have indicated that in high‐grade bladder cancer, BAMBI expression may be regulated by epigenetic mechanisms such as DNA methylation [35]. The above studies indicate that BAMBI expression is regulated by both transcriptional and epigenetic mechanisms in various types of cancers.

In the broader context of integrating single‐cell sequencing with machine learning–based computational strategies, our study provides a focused example of how high‐dimensional single‐cell transcriptomic data can be used to dissect tumor heterogeneity and prioritize metastasis‐associated biomarkers in osteosarcoma. By combining unsupervised clustering, inferCNV‐based malignant cell inference, pseudotime analysis, SCENIC regulatory network analysis, JASPAR motif prediction, external dataset validation, and in vitro functional assays, we identified a metastasis‐related JUN–BAMBI regulatory axis that may link malignant osteoblastic cell‐state transition with TGF‐*β* pathway activation. Although a formal supervised machine learning prediction model was not constructed in the present study, several analytical steps used here reflect the logic of computational feature selection and pattern recognition in single‐cell data. Future studies incorporating larger single‐cell cohorts, spatial transcriptomics, multiomics data, and supervised learning algorithms may further validate BAMBI, JUN regulon activity, CNV burden, TGF‐*β* pathway activity, and immune‐cell composition as integrated features for predicting metastatic risk, treatment response, or recurrence in osteosarcoma. Such efforts would help translate single‐cell discoveries into reproducible biomarkers and clinically applicable decision‐support tools for precision oncology.

This study combined bioinformatics analyses and in vitro functional experiments to investigate the regulatory role of BAMBI in the migration and invasion process of OS cells. The results demonstrated a negative correlation between BAMBI expression levels and the migration and invasion potential across different OS cell lines. Specifically, cell lines with high migration and invasion capability exhibited relatively low BAMBI expression. BAMBI likely inhibits OS cell migration and invasion by suppressing the TGF‐*β*–induced EMT signaling pathway. Although this study combined single‐cell transcriptomic analysis with in vitro functional experiments to preliminarily reveal the inhibitory role of BAMBI in OS invasion and metastasis and suggested that its expression may be regulated by the upstream transcription factor JUN, certain limitations remain. First, the number of single‐cell RNA sequencing samples included in this study was relatively limited, and the sample sources were restricted, which may affect the representativeness of the findings. However, the incorporation of an independent validation dataset partially mitigates this limitation. In addition, although JASPAR‐based motif prediction and analysis of the independent validation dataset further support a potential regulatory association between JUN and BAMBI, direct evidence of transcriptional regulation, such as chromatin immunoprecipitation (ChIP) assays, is still lacking. Therefore, the relationship between JUN and BAMBI cannot be conclusively defined as a direct regulatory interaction. Moreover, key components of the TGF‐*β* signaling pathway and EMT were not experimentally evaluated in this study, which limits the completeness of the proposed mechanistic framework. Future studies should incorporate larger scale single‐cell datasets, in vivo functional experiments, and more direct mechanistic investigations to further elucidate the role of the JUN/BAMBI/TGF‐*β* axis in osteosarcoma progression and metastasis. From a translational perspective, BAMBI may have potential as a biomarker for metastatic risk and as a therapeutic target in osteosarcoma. Further validation in larger clinical cohorts, particularly to assess its association with metastasis and patient prognosis as well as exploration of therapeutic strategies targeting BAMBI or related TGF‐*β* signaling pathways, may provide new insights into precision treatment of OS.

## 5. Conclusion

This study identifies BAMBI as a negative regulator of osteosarcoma metastasis. Single‐cell transcriptomics revealed specific downregulation of BAMBI in metastasis‐associated osteoblast clusters, which was linked to activation of the TGF‐*β* signaling pathway. Functional assays confirmed that BAMBI overexpression suppressed, whereas knockdown enhanced, osteosarcoma cell migration and invasion. JUN was predicted as an upstream regulator, suggesting a potential “JUN–BAMBI–TGF‐*β*” axis. These findings indicate that BAMBI may serve as a biomarker for metastatic risk and a therapeutic target, supporting further validation in larger cohorts and clinical settings.

## Author Contributions

**Ning Song:** conceptualization, software, writing – original draft. **Qiang Zhang:** validation, formal analysis, data curation, writing – review and editing. **Junwei Du:** data curation, writing – review and editing. **Renbing Jiang:** resources, writing – review and editing, funding acquisition.

## Funding

This study received no financial support.

## Ethics Statement

The authors have nothing to report.

## Conflicts of Interest

The authors declare no conflicts of interest.

## Supporting information


**Supporting Information** Additional supporting information can be found online in the Supporting Information section. Table S1: siRNA sequences used for gene knockdown. Table S2: The primer sequences for qRT‐PCR reactions. Figure S1: inferCNV analysis reveals increased copy number variation in metastatic osteosarcoma osteoblasts. Figure S2: The identification of cell types in the GSE152048 dataset. Figure S3: Single‐cell analysis of osteoblast subclusters in osteosarcoma. Figure S4: Boxplot comparing JUN expression levels between metastatic and nonmetastatic cell clusters.

## Data Availability

The data that support the findings of this study are available in Gene Expression Omnibus at https://www.ncbi.nlm.nih.gov/geo/ (Reference Numbers GSE250015, GSE152048). These data were derived from the following resources available in the public domain: GSE250015 (https://www.ncbi.nlm.nih.gov/geo/query/acc.cgi?acc=GSE250015) and GSE152048 (https://www.ncbi.nlm.nih.gov/geo/query/acc.cgi?acc=GSE152048).
