# Supplementary material for: Single‐Cell Transcriptomic Analysis Suggests That JUN May Regulate BAMBI to Promote Osteosarcoma Cell Migration and Invasion
Source: Hum Mutat. 2026 May 27;2026:9261651. doi: 10.1155/humu/9261651 (PMC13214976; doi:10.1155/humu/9261651)
Supplement: Supplementary file 1 — Supporting Information Additional supporting information can be found online in the Supporting Information section. Table S1: siRNA sequences used for gene knockdown. Table S2: The primer sequences for qRT‐PCR reactions. Figure S1: inferCNV analysis reveals increased copy number variation in metastatic osteosarcoma osteoblasts. Figure S2: The identification of cell types in the GSE152048 dataset. Figure S3: Single‐cell analysis of osteoblast subclusters in osteosarcoma. Figure S4: Boxplot comparing JUN expression levels between metastatic and nonmetastatic cell clusters. [file HUMU-2026-9261651-s001.docx]

Supplementary Material

Supplementary Tables

**Supplementary Table 1. siRNA sequences used for gene knockdown**

| Primer name | Forward (5’-3’) | Reverse (5’-3’) |
| --- | --- | --- |
| BAMBI(homo-ID:25805)siRNA-1- 772 | GCAGAAACCUUAUCACCAATT | UUGGUGAUAAGGUUUCUGCTT |
| BAMBI(homo-ID:25805)siRNA-2- 1052 | CUGUCUGACCUGUGAUAAATT | UUUAUCACAGGUCAGACAGTT |
| BAMBI(homo-ID:25805)siRNA-3-916 | AUAAGAGGCUGCAGGAUCATT | UGAUCCUGCAGCCUCUUAUTT |
| si-NC(Negative control) | UUCUCCGAACGUGUCACGUTT | ACGUGACACGUUCGGAGAATT |
| Si-GAPDH(Positive control ) | GUAUGACAACAGCCUCAAGTT | CUUGAGGCUGUUGUCAUACTT |
| JUN（human）ID:3725-siRNA-1732 | GGAUCAAGGCGGAGAGGAATT | UUCCUCUCCGCCUUGAUCCTT |
| JUN（human）ID:3725-siRNA-1896 | CAGCUUAAACAGAAAGUCATT | UGACUUUCUGUUUAAGCUGTT |
| JUN（human）ID:3725-siRNA-1048 | GACCUUAUGGCUACAGUAATT | UUACUGUAGCCAUAAGGUCTT |
| si-NC(Negative control) | UUCUCCGAACGUGUCACGUTT | ACGUGACACGUUCGGAGAATT |
| Si-GAPDH(Positive control ) | GUAUGACAACAGCCUCAAGTT | CUUGAGGCUGUUGUCAUACTT |

**Supplementary Table 2.** The primer sequences for qRT-PCR reactions

| Gene name | Forward (5’-3’) | Reverse (5’-3’) |
| --- | --- | --- |
| BAMBI(human) | TACAGAGGGCTGCACGATGTTC | AAGTCAGCTCCTGCACCTTGGT |
| JUN(human) | CCTTGAAAGCTCAGAACTCGGAG | TGCTGCGTTAGCATGAGTTGGC |
| β-actin (human) | TCCTTCCTGGGCATGGAGT | AGCACTGTGTTGGCGTACAG |

# Supplementary Figures


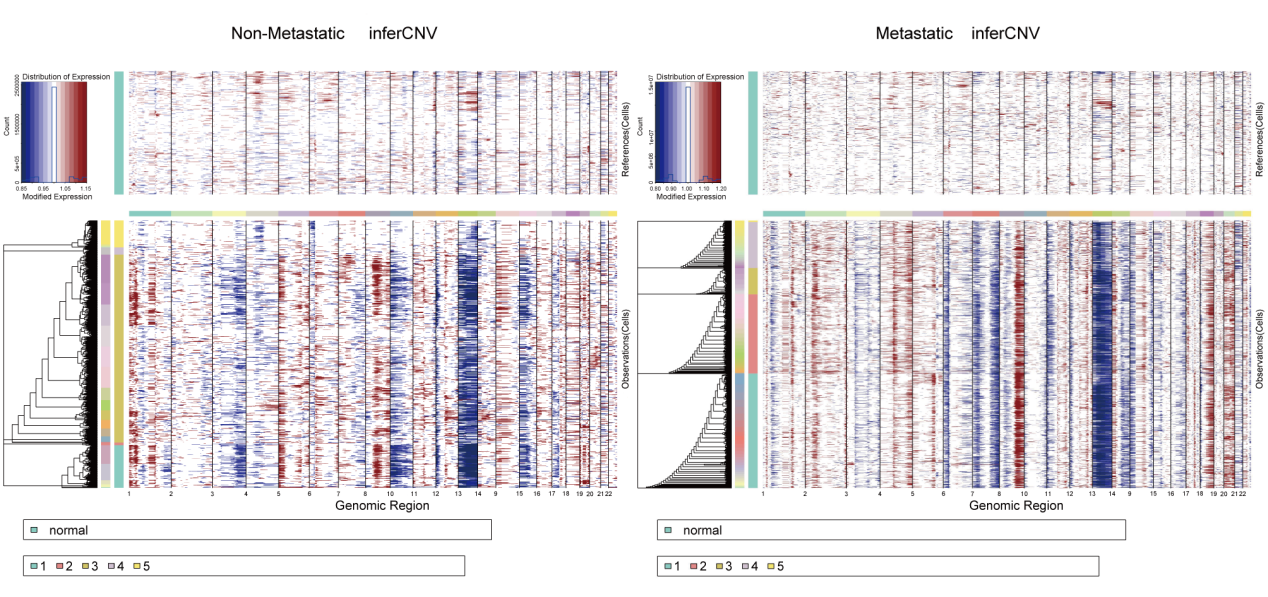


**Supplementary Figure 1.** inferCNV analysis reveals increased copy number variation in metastatic osteosarcoma osteoblasts. Using inferCNV, we inferred CNV in osteosarcoma osteoblasts, and the results indicated that metastatic cells exhibited more extensive and pronounced CNV alterations compared with non-metastatic cells.


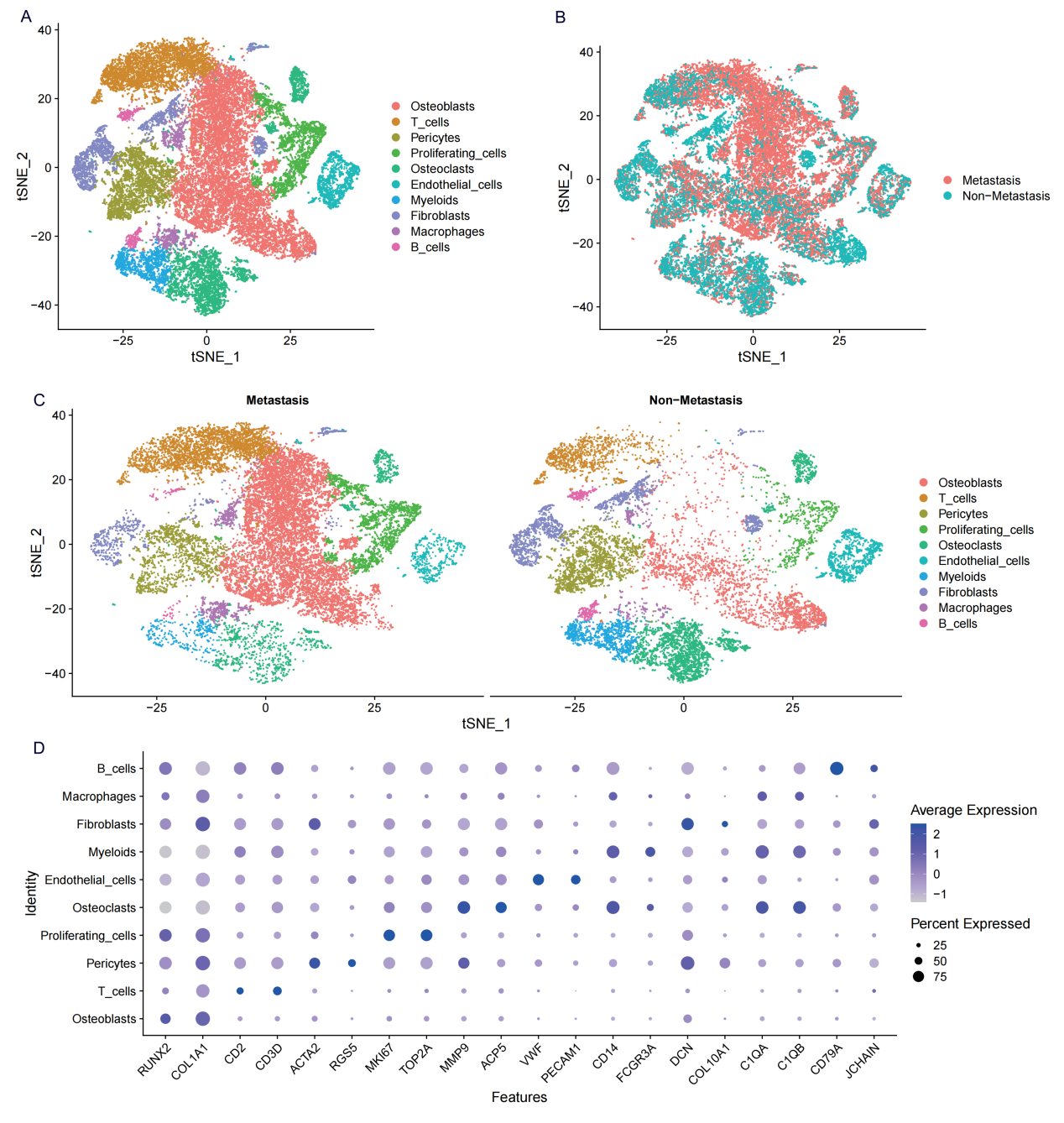


**Supplementary Figure 2.** The identification of cells types in GSE152048 dataset. **(A)** t-SNE plot showing ten distinct cell clusters identified in osteosarcoma samples; **(B)** overall t-SNE visualization illustrating both metastatic and non-metastatic osteosarcoma lesions; **(C)** comparison of the distribution patterns of the ten clusters between metastatic and non-metastatic lesions; **(D)** expression profiles of signature genes across the ten clusters.


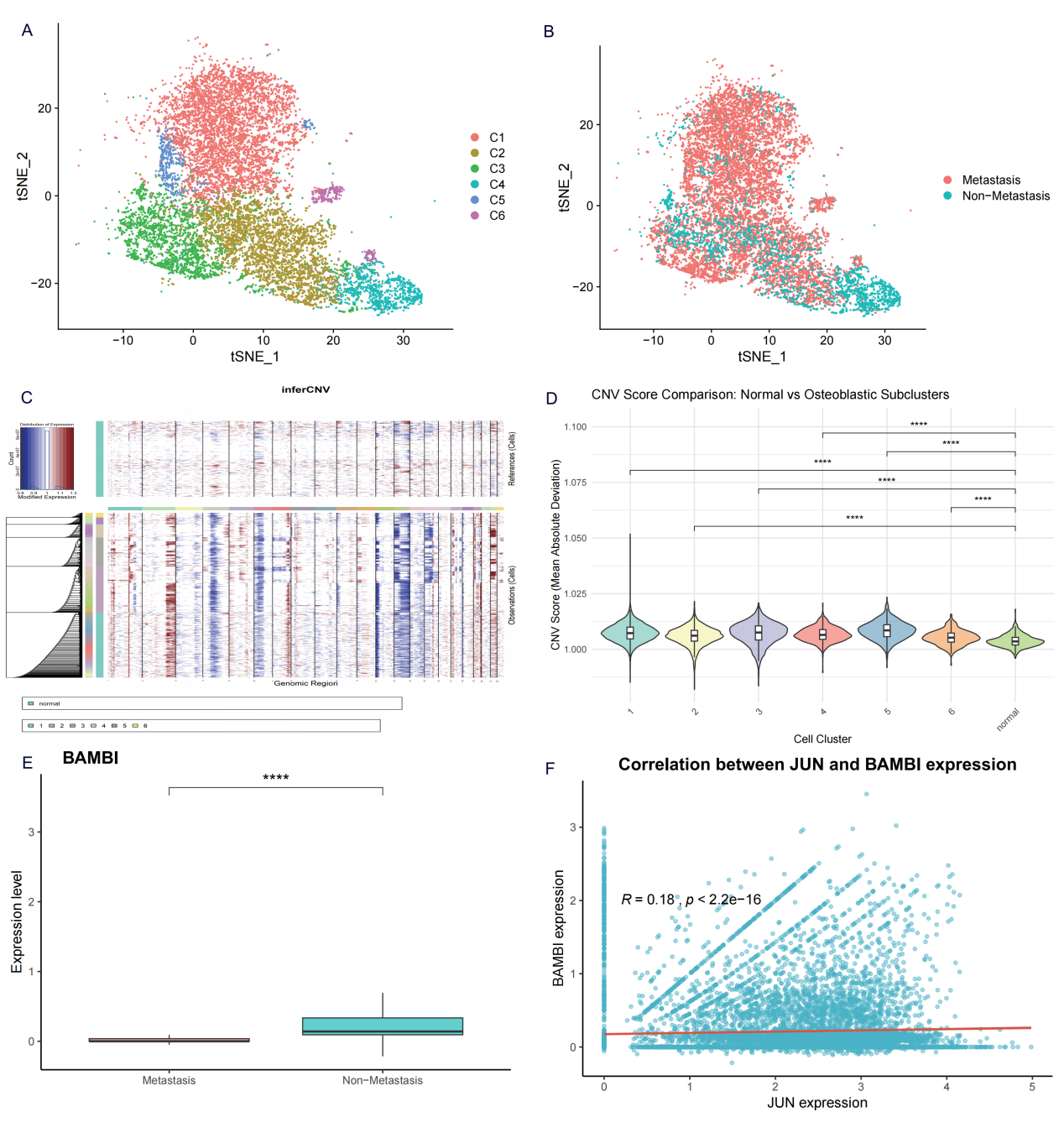


**Supplementary Figure 3.** Single-cell analysis of osteoblast subclusters in osteosarcoma. **(A)** t-SNE analysis identifying six major osteoblast subclusters; **(B)** t-SNE plot showing osteoblasts from metastatic and non-metastatic osteosarcoma lesions; **(C)** Heatmap illustrating CNV profiles of osteoblast subclusters; **(D)** Boxplot comparing CNV scores between endothelial cells and osteoblast subclusters; **(E)** Boxplot comparing BAMBI expression levels between metastatic and non-metastatic cell clusters; **(F)** Correlation analysis between JUN and BAMBI expression.


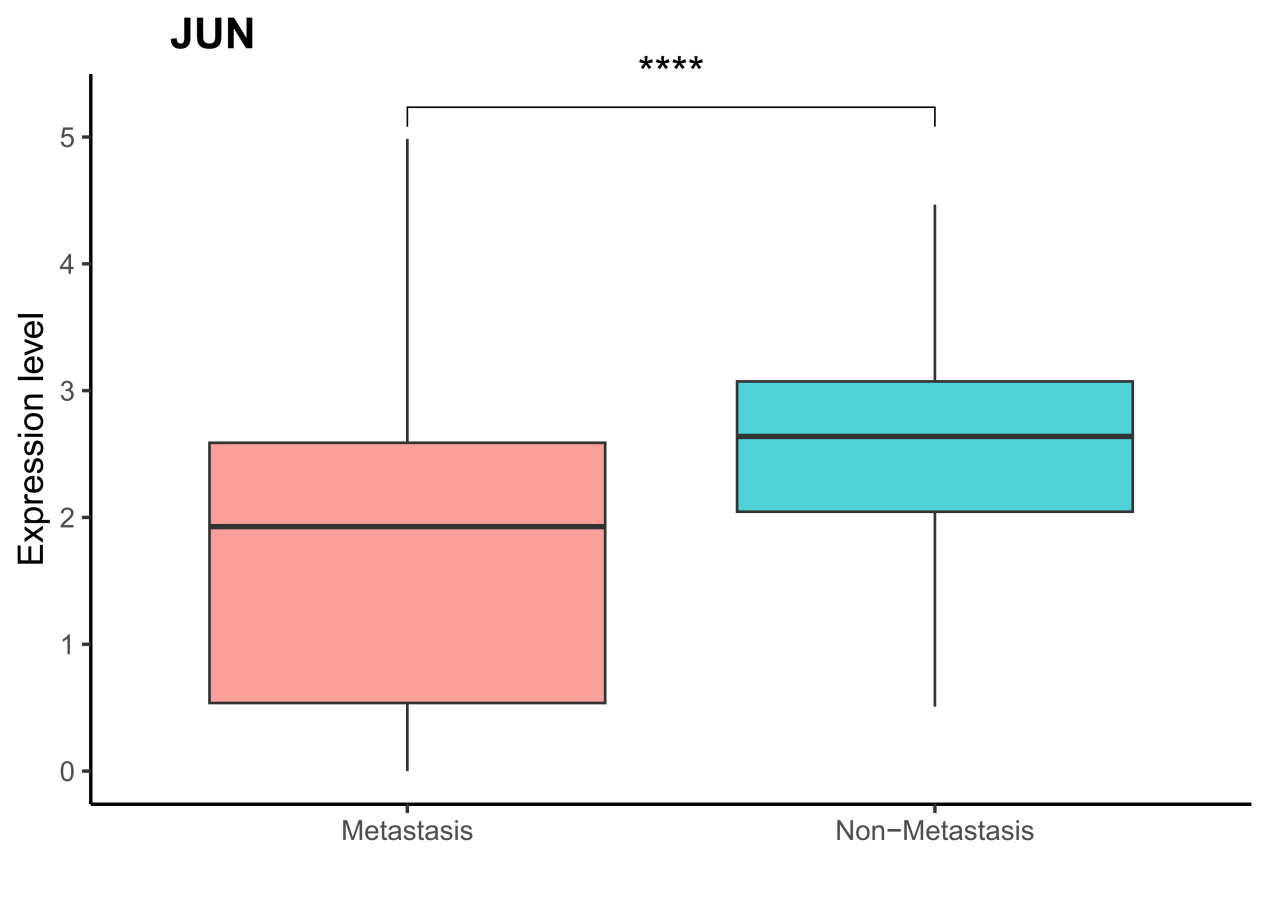


**Supplementary Figure 4.** Boxplot comparing JUN expression levels between metastatic and non-metastatic cell clusters.
